# Potential antidiabetic phytochemicals in plant roots: a review of in vivo studies

**DOI:** 10.1007/s40200-021-00853-9

**Published:** 2021-07-12

**Authors:** Hamidreza Ardalani, Fatemeh Hejazi Amiri, Amin Hadipanah, Kenneth T. Kongstad

**Affiliations:** 1 Department of Drug Design and Pharmacology, Faculty of Health and Medical Sciences, University of Copenhagen, Universitetsparken 2, 2100 Copenhagen, Denmark; 2 Department of Chemistry, Centre for Analysis and Synthesis, Lund University, Lund, Sweden; 3 Department of Microbiology, Faculty of Medicine, Babol University of Medical Sciences, Babol, Iran; 4 Department of Plant Biology, Faculty of Sciences, Shahrekord University, Shahrekord, Iran

**Keywords:** Diabetes, Medicinal plant, Natural product, α-glucosidase, Phytochemical, In vivo

## Abstract

**Background:**

Medicinal plants are used to treat various disorders, including diabetes, globally in a range of formulations. While attention has mainly been on the aerial plant parts, there are only a few review studies to date that are focused on the natural constituents present in the plant roots with health benefits. Thus, the present study was performed to review in vivo studies investigating the antidiabetic potential of the natural compounds in plant roots.

**Methods:**

We sorted relevant data in 2001–2019 from scientific databases and search engines, including Web of Knowledge, PubMed, ScienceDirect, Medline, Reaxys, and Google Scholar. The class of phytochemicals, plant families, major compounds, active constituents, effective dosages, type of extracts, time of experiments, and type of diabetic induction were described.

**Results:**

In our literature review, we found 104 plants with determined antidiabetic activity in their root extracts. The biosynthesis pathways and mechanism of actions of the most frequent class of compounds were also proposed. The results of this review indicated that flavonoids, phenolic compounds, alkaloids, and phytosteroids are the most abundant natural compounds in plant roots with antidiabetic activity. Phytochemicals in plant roots possess different mechanisms of action to control diabetes, including inhibition of *α*-amylase and *α*-glucosidase enzymes, oxidative stress reduction, secretion of insulin, improvement of diabetic retinopathy/nephropathy, slow the starch digestion, and contribution against hyperglycemia.

**Conclusion:**

This review concludes that plant roots are a promising source of bioactive compounds which can be explored to develop against diabetes and diabetes-related complications.

**Graphical abstract:**

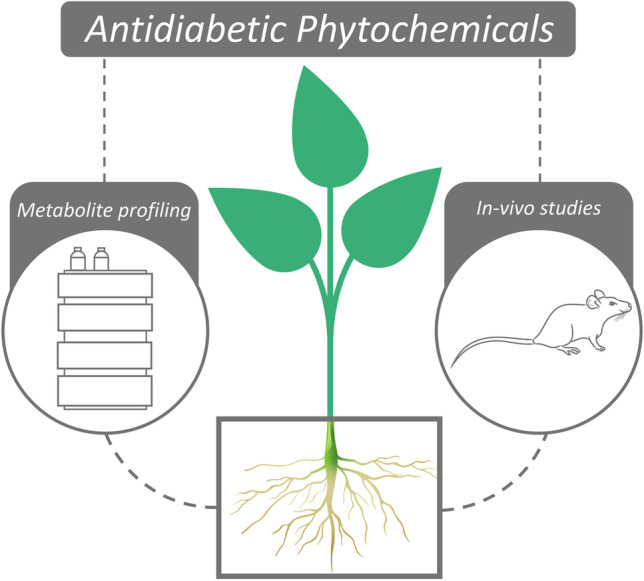

## Introduction

A recent analysis of the prevalence of diabetes mellitus, with type 2 diabetes (T2D) being the dominant form, estimated 4.2 million deaths worldwide due to diabetes in 2019. The direct medical cost for treatment of this metabolic disorder was estimated at 760 billion U.S. dollars, corresponding to 10% of the total health care expenses [1]. The common risk factors for developing T2D are obesity and lacking exercise. With a worldwide general obesity epidemic, the projected numbers of individuals with T2D are expected to increase dramatically from 463 million in 2019 to 700 million in 2045, highlighting the need for efficient drugs for managing T2D [1]. Weight-reduction and lifestyle improvements, such as the increase in physical activity and intake of functional foods (i.e., foods with health-promoting effects beyond their nutritional values), are effective methods for controlling blood glucose levels, alleviating some of the T2D complications [2, 3]. Pharmaceutical methods for the treatment of T2D include metformin, which can reduce 30% of the T2D progression even without lifestyle changes, at the cost of possible side effects such as vitamin B12 deficiency [2, 4]. Generally, T2D is manifested by decreased insulin-stimulated glucose uptake by the skeletal muscles. The resulting low peripheral glucose disposition and high hepatic glucose production are primary contributors to diabetic hyperglycemia, leading to micro- and macro-vascular complications, including retinopathy, neuropathy, nephropathy, cardiovascular disease, stroke, and amputations [5–8]. The existing clinical agents targeting these complications, such as acarbose, voglibose, and miglitol, are associated with gastrointestinal side effects such as nausea, constipation, and diarrhea due to the nature of their mechanism of action [9]. Thus, alternative agents with fewer side effects, such as natural products derived from plants and microorganisms, are in demand for future T2D management. In addition, the increased incidence of diabetes calls for the development of useful and novel therapy procedures. Plant-based remedies, in the forms of teas, capsules, extracts, or isolated phytochemicals, are commonly used as complementary therapies to control T2D complications [10]. Different plant parts often exhibit distinctive chemical profiles contributing to antidiabetic bioactivities. Alkaloids, flavonoids, phytosteroids, and phenols are the most abundant compound classes with demonstrated antidiabetic effects in plant roots [11, 12].

Plants have always been an outstanding source of food, drug, and recent numbers show that more than 45% of all approved drugs from 1981 to 2019 are of natural origin or mimics thereof [13]. With accelerated improvements in novel analytical techniques [14, 15] and an increase in the number of studies on natural products with antidiabetic bioactivity, a range of new compounds from various unique plants has been found to possess antidiabetic activities [16]. While existing reviews predominantly focus on the antidiabetic bioactivity of the aerial plant parts, there is limited knowledge of in vivo antidiabetic effects of natural constituents present in the plant roots and rhizomes.

Thus, the main aim of this review was to summarize the potential antidiabetic natural products in plant roots and rhizomes with emphasis on in vivo effects.

## Methods

To build and collect data for this review, several databases and search engines, including Web of Knowledge, PubMed, Science Direct, Medline, Reaxys, and Google Scholar were used. The used keywords were included: “medicinal plant roots”, “antidiabetic natural products”, “diabetic rats”, “in vivo studies”, and “herbal medicine”. In vitro studies and investigations that did not concern root and rhizomes were excluded. The search was limited to studies in English, and the dates of the studies ranged from 2001 to 2019.

## Results and discussion

In the past decades, people have used different parts of medicinal plants as antidiabetic remedies. Recently, several traditional plant-based treatments have been reported to manage diabetes, according to in vitro, in vivo*,* and clinical investigations. Plant roots contain a diverse range of phytochemicals such as flavonoids, phenols, alkaloids, tannins, phytosterol, and saponins [17], with studies showing that some compounds are being uniquely biosynthesized in the root system [18–20].

**Fig. 1:**
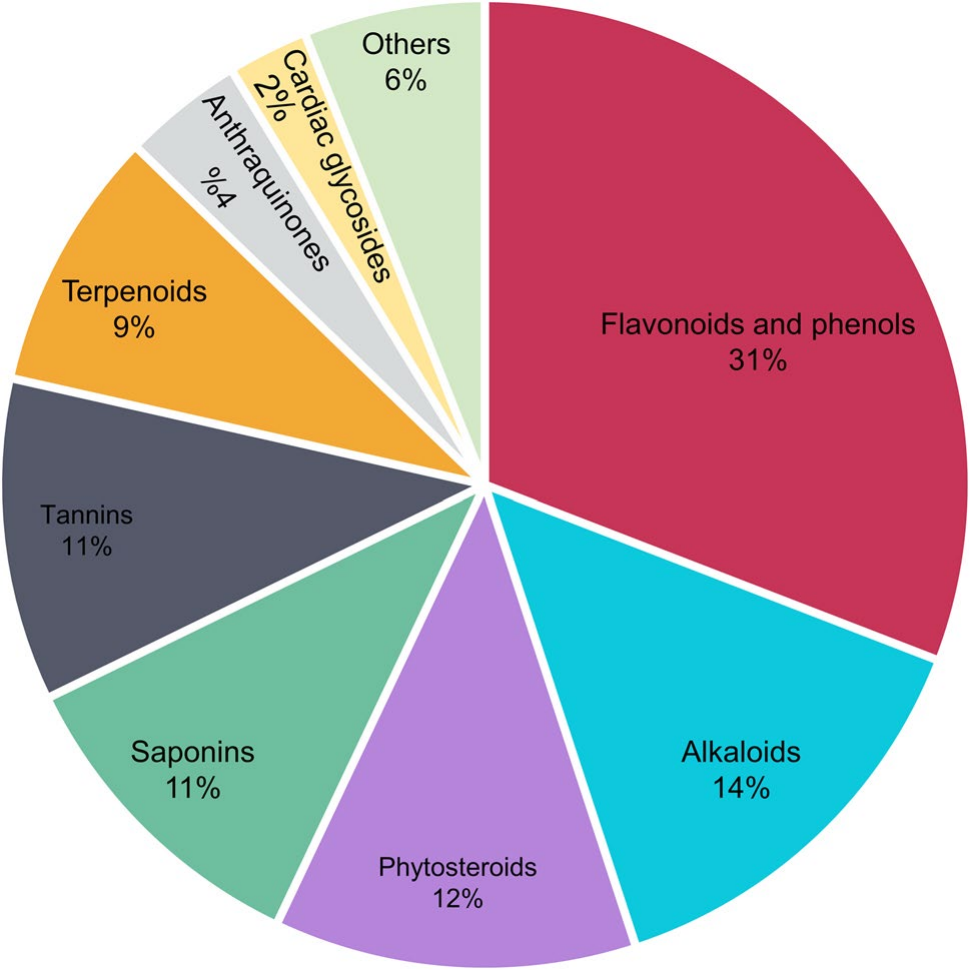
The class of compounds with antidiabetic bioactivity in plant roots and rhizomes

**Fig. 2:**
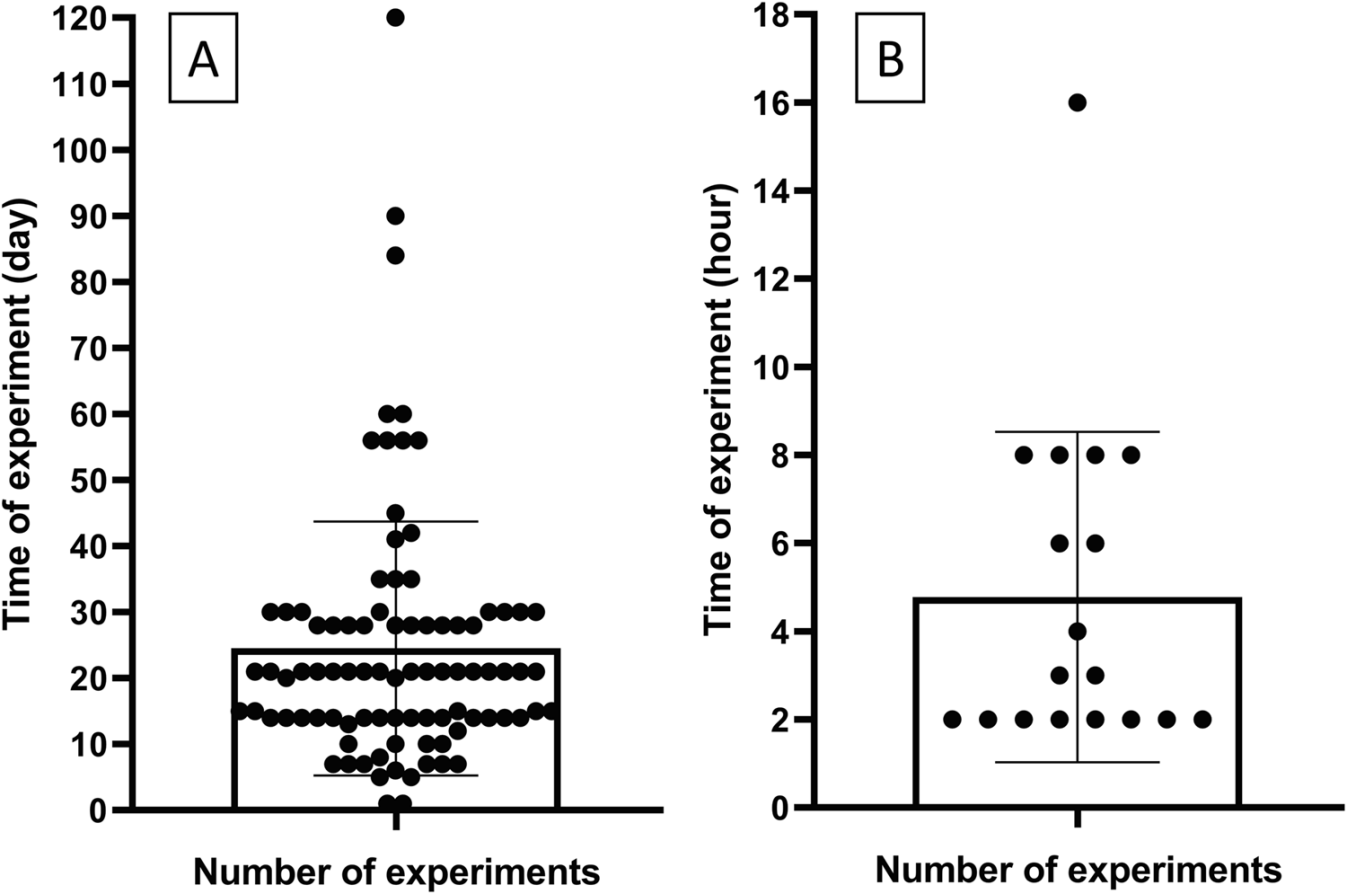
The time of experiments in the reviewed in vivo studies. A: long-time (more than one day, n: 90), B: short-time (less than one day, n: 18)

From the literature review, a total of 104 plant species from 56 families were found to contain antidiabetic compounds in their roots and rhizomes (Table 1). The most frequent plant families which were reported in the reviewed studies were Fabaceae, Araliaceae, Asparagaceae, Asteraceae, and Zingiberaceae, respectively. While not all reviewed studies report the chemical constituents or bioactive compounds, the results showed that flavonoids and phenols, alkaloids, phytosteroids, saponins, tannins, terpenoids, anthraquinones, and cardiac glycosides were the most abundant bioactive components in plant roots and rhizomes (Fig. 1) as described in detail below. In the reviewed studies, a range of solvents was used for the extraction of natural constituents. The most common were ethanol (28%), water (27%), and methanol (22%). The time of experiments varied among the studies from 2 h to 120 days. Therefore, we categorized the time of experiments into two categories: short time (less than one day) and long-time experiments (more than one day). The results showed that 17% of the experiments were performed within a day (short time), while 83% of the experiments were performed in more than one day (long time). The average time of the two categories were 5 h and 25 days, respectively (Fig. 2).

**Table 1:** The list of plants with antidiabetic activity in their roots and rhizomes extracts

Scientific name	Common name	Family	Major chemical constituents	Bioactive compound	Extract type	Dose (mg/kg)	Effective dose (mg/kg)	Time (days)	Induction of diabetes	Experimental animals	References
*Acanthus ilicifolius*	Sea holly	Acanthaceae	Flavonoids, alkaloids, terpenoids, tannins, phytosteroids	-	Ethanolic	200, 400	≥ 200	14	Alloxan	Male albino Wistar rat	[21]
*Acorus calamus* L	Sweet flag or calamus	Acoraceae	-	-	Ethyl acetate	100	100	28 for STZ induced and 35 for db/db	Streptozotocin	Male mice	[22]
*Actinidia kolomikta* (Maxim. et Rur.) Maxim	Variegated kiwi vine	Actinidaceae	Polyphenols	-	Ethanolic	300	300	0.1	-	Male Sprague–Dawley rats	[23]
			Flavonoids			100,200,400	400				
*Aerva lanata* (L.) Juss. ex Schult	Knotgrass	Amaranthaceae	Alkaloids	Canthin-6-one derivatives	Methanolic	10, 20	-	15	Streptozotocin-nicotinamide	Male–female wistar albino rats	[24]
*Alpinia calcarata*	Snap ginger or cardamom ginger	Zingiberaceae	**-**	-	Ethanolic	200	200	30	Streptozotocin	Albino rats	[25]
*Alpinia galanga* L	Greater galangal	Zingiberaceae	Alkaloids, saponins, glycosides, flavonoids, phytosteroids, terpinoids	-	Ethanolic	200, 400	≥ 200	21	-	Wistar rats	[26]
*Anacyclus pyrethrum* DC	Pellitory or Akarkara	Asteraceae	Flavonoids	-	Aqueous	150, 300	≥ 150	0.1	Alloxan	Albino wistar rats	[27]
*Andrographis paniculata* (Burm.f.) Nees	Creat or Green chirera	Acanthaceae	-	-	Chloroform	50, 100, 150	≥ 50	1	Alloxan	Sprague–Dawley rat	[28]
						150	150	28			
*Anemarrhena asphodeloides* Bunge	Zhi Mu	Asparagaceae	-	Mangiferin, mangiferin-7-*O*-β-glucoside	Aqueous	90	90	0.3	-	KK-Ay Mice	[29]
*Anthocleista djalonensis* A. Chevalier	Tagare, foreta lafira	Loganiaceae	Flavonoids, saponins, tannins, cardiac glycosides, anthraquinones	-	Ethanolic	37, 74, 111	≥ 37	14	Alloxan	Swiss albino mice and rats	[30]
					Chloroform	74	74				
					Ethyl acetate	74	74				
					Methanolic	74	74				
*Anthocleista vogelii* (Planch)	Cabbage tree	Gentianaceae	Flavonoids, terpenes, phenols, lipids, alkaloids,fatty acids	Quebrachitol, loganin, sweroside, oleoside 11-methyl ester and ferulic acid	Methanolic, chloroform	100,200	-	(acute study)0.1 day study)21 days	Alloxan	Male Sprague–Dawley rats	[31]
*Aporosa lindleyana* (wt.) Bail	Kotili	Euphorbiaceae	-	-	Alcoholic	100	-	0.1	Alloxan	Male Albino wistar Rats	[32]
*Aralia elata*	Angelica-tree,Taranoki	Araliaceae	-	-	Aqueous	125	-	0.1	-	Male ddy mice	[33]
*Aralia taibaiensis*	Spikenard	Araliaceae	Triterpenoids, saponins	28-*O*-β-D-glucopyranosyl ester	Alcoholic	75,150,300	≥ 75	28	Streptozotocin	Male Albino wistar rats	[34]
*Artocarpus communis* Forst	Breadfruit,Gbere	Moraceae	-	-	Aqueous	100	100	7	Streptozotocin	Wistar rats	[35]
*Asparagus racemosus* (Wild)	Shatavari	Asparagaceae	-	-	Ethanolic	200, 400	≥ 200	21	Streptozotocin	Wistar rats	[36]
*Atractylodes japonica* Koidz	Japanese atractylodes	Asteraceae	-	-	-	100	100	28	High fat diet and Streptozotocin	Sprague–Dawley rats	[37]
*Azadirachta indica* A.Juss	Neem	Meliaceae	-	-	Alcoholic	200, 400, 800	800	15	Alloxan	Albino wistar rats	[38]
*Berberis aristata* DC	Daruharidra	Berberidaceae	-	Berberine, berbamine, palmatine	Aqueous, Ethanolic	250	-	21	Alloxan	Male albino wistar rats	[39]
*Berberis lyceum* Royle	Indian barberry	Berberidaceae	-	-	Aqueous	50, 100	≥ 50	5	Alloxan	Wistar rats	[40]
					Ethanolic						
*Berberis vulgaris* L	Barberry	Berberidaceae	Tannins, alkaloids, saponins, phytosteroids, anthraquinones	-	Aqueous	25	25	21	Streptozotocin	Male Wistar rats	[41]
					Alcoholic	62.5	62.5				
*Boerhavia diffusa* L	Punarnava,spreading hogweed,tarvine	Nyctaginaceae	Phenols, flavonoids	Gallic acid, quercitin	Methanolic	200		7	Streptozotocin	Male Wistar rats	[42]
*Brassica rapa* L	Turnip	Brassicaceae	Flavonoids, polyphenols	-	Ethanolic	2600	2600	35	-	Db/db mice	[43]
*Bruguiera gymnorrhiza* L	Black mangrove or afrikaans	Rhizophoraceae	Alkaloids, phytosteroids, saponins	-	Ethanolic	400	400	21	Streptozotocin	Rats	[44]
*Caesalpinia digyna* Rottler	Teri pods or udakiryaka	Fabaceae	-	Bergenin	Ethanolic	2.5, 5, 10	10	14	Streptozotocin-Nicotinamide	Male albino rats	[45]
*Cajanus cajan* L	Arhar(Pigeon pea)	Fabaceae	Phenols	-	Methanolic	200, 400	≥ 200	5	Alloxan	Swiss albino mice	[46]
*Casearia esculenta* (Roxb.)	Kadala zhinjill,wild cowrie fruit,saptarangi	Flacourtiaceae	-	-	Aqueous	200,300		45	Streptozotocin	Male albino rats	[47]
*Ceiba pentandra* L	Silk cotton tree	Sterculiaceae	-	-	Ethanolic	300	300	30	alloxan	Male Wistar rat	[48]
*Cichorium intybus*	Chicory	Asteraceae	Inulin, lipids, alkaloids, glycosides, tannins	-	Methanolic	400	400	21	Streptozotocin	Male, Wistar albino rats	[49]
*Citrullus colocynthis*	Bitter cucumber,Bitter apple,egusi	Cucurbitaceae	Glycosides, saponins, triterpenoids, alkaloids, flavonoids, resins	-	Aqueous	200	200	7	Alloxan	Male Wistar rats	[50]
					Chloroform	200	-				
					Ethanolic	200	-				
*Clausena anisata* (Willd) Hook	Isifudu	Rutacaea	-	-	Methanolic	100–800	≥ 800	-	Streptozotocin	Male Wistar rats	[51]
*Coptis chinensis Franch*	Goldthread	Ranunculaceae	Alkaloids	Berberine, palmatine, jatrorrhizine	Aqueous	125,250,500	≥ 125	21	Alloxan	Wistar rats	[52]
*Costus speciosus* (Koen.) Sm	Crepe ginger	Costaseae	-	-	Hexane	250	250	60	Streptozotocin	Wistar rats	[53]
					Ethyl acetate	400	400				
					Methanolic	400	400				
*Curculigo orchioides* Gaertn	Talamuli,musali, nilapanai	Hypodoxiaceae	-	-	Ethanolic	500, 1000	≥ 500	21	Alloxan	Swiss albino mice	[54]
					Aqueous	500,1000					
*Curcuma aromatica*	Turmeric	Zingiberaceae	Phenols, flavonoids, flavonols	-	Toluene	200, 400	≥ 200	21	Streptozotocin	Wister albino rats	[55]
*Curcuma longa*	Turmeric	Zingiberaceae	-	-	Aqeous	400	400	28	Alloxan	Albino rats	[56]
					Methanolic	400	400				
					Hexane	400	400				
*Cyperus rotundus* L	Mustaka	*Cyperaceae*	-	-	Ethanolic	250, 500	≥ 250	21	Streptozotocin	Swiss albino mice	[57]
*Datura stramonium* L	Jimsonweed	Solanacaea	Flavonoids, phenols, tannins, alkaloids, phytosteroids, glycosides, and anthraquinones	-	Methanolic	100, 200, 400	≥ 100	14	Streptozotocin	Swiss albino mice	[58]
*Dioscorea dumetorum* Pax	Bitter yam or cluster yam	Dioscoreaceae	Flavonoids, alkaloids, saponins, cardiac glycosides	-	Aqueous	400	400	7	Alloxan	Albino Wistar rats	[59]
*Elephantopus scaber*	Elephant’s foot	Asteraceae	**-**	-	Methanolic	250	-	60	Streptozotocin	Male Albino Wistar rats	[60]
					Ethyl acetate	250	250				
					Hexane	250	-				
*Euclea undulata* Thunb. var. myrtina	Guarri	Ebenaceae	-	-	Acetone	50, 100	100	21	Streptozotocin-nicotinamide	Male Wistar rats	[61]
*Glycyrrhiza glabra*	Licorice	Fabaceae	-	-	Methanolic	100,200,300	≥ 200	0.1	Streptozotocin	Albino rats	[62]
*Glycyrrhiza uralensis* Fisch	Licorice	Fabaceae	-	Glycyrrhizin, glycyrrhetinic acid	Ethanolic	1	1	56	-	Male C57BL6J mice	[63]
*Gmelina asiatica* L	Nilakkumil or gopabhandra	Verbenaceae	-	-	Alcoholic	100, 250, 500	≥ 100	16 h	Alloxan	Sprague Dawley rats	[64]
*Gynandropsis gynandra*	Shona cabbage or African cabbage	Capparidaceae	Flavonoids, phenolic compounds, glycosides, phytosteroids, phenolic	-	Aqueous	100, 200, 400	≥ 100	0.7	Streptozotocin	Albino rats	[65]
*Harpagophytum procumbens* DC	Devil’s claw or grapple plant	Pedaliaceae	-	-	Aqueous	50,100,200,400,800	-	0.3	Streptozotocin	Wistar rat	[66]
*Helicteres isora* L	Screw tree	Sterculiaceae	Triterpenoidal glycosides	-	Butanolic	250	250	10	Alloxan	Male Wistar rats	[67]
					Ethanolic	250	250				
*Hemidesmus indicus* R.Br	Indian sarsaparilla	Asclepiadaceae	Flavonoids, alkaloids, saponins, triterpenoids, tannins, phytosteroids, phenols	-	Methanolic	200, 400	400	90	Streptozotocin	Albino Wistar rat	[68]
*Ibervillea sonorae*	Wareque	Cucurbitaceae	Phenols, phytosteroids	-	Dichloromethane, methanolic	300, 600	≥ 300	41	Alloxan	Wistar rats	[69]
*Ichnocarpus frutescens* (L.) R.Br	Black creeper or dudhilata	Apocynaceae	-	-	Aqueous	250, 500	≥ 250	15	Streptozotocin-nicotinamide	Male albino Wistar rats	[70]
*Ipomoea batatas* L	Sweet potato	Convolvulaceae	-	-	Methanolic	4000	-	14	Alloxan	Male Wistar rats	[71]
*Justicia adhatoda* L	Malabar nut	Acanthaceae	-	-	Ethanolic	100	100	6	Alloxan	Wistar rats	[72]
*Liriope spicata* var. prolifera	Creeping lilyturf & monkey grass	Liliaceae	-	-	Aqueous	100, 200	≥ 100	28 (FBS)	Streptozotocin	Male BABL/c mice	[73]
								14(OGTT)			
*Lycii radices* or *Lycium chinense* Miller	Goji berry or wolfberry	Solanaceae	-	-	Aqueous	80, 160	- (in serum)	14	Streptozotocin	Male Sprague–Dawley rats	[74]
							≥ 80(in kidney)				
*Merremia tridentata* (L.) Hall. F	Mudiarkunthal or savulikodi,Thrippanpullo	Convolvulaceae	-	-	Aqueous	50, 100, 150	≥ 50	21	Streptozotocin	Male albino Wister rats	[75]
*Mimosa pudica*	Sensitive plant, humble plant, Lajwanti	Fabaceae	-	-	-	2, 4, 6	6	20	Alloxan	Albino rabbits	[76]
*Morus alba* L	Mulberry tree	Moraceae	Flavonoids, terpenoids	Morusin, cyclomorusin, neocyclomorusin, kuwanon E, 2-arylbenzofuran, moracin M betulinic acid, methyl ursolate	Ethanolic	200,400,600	600	10	Streptozotocin	Male Wister rats	[77]
*Musa paradisiaca* L	Banana	Musaceae	-	-	Methanolic	800	800	14	Streptozotocin	Male albino rats	[78]
*Nauclea latifolia* Sm	Pin cushion tree	Rubiaceae	Tannins, saponins, alkaloids, terpenes, cardiac glycosides, flavonoids, anthraquinones	-	Ethanolic	150, 300, 450	≥ 450	14	Alloxan	Swiss albino mice and rats	[79]
*Nyctanthes arbor-tristis* L	Harsinghar or night jasmine	Oleaceae	-	-	Methanolic	250, 500	≥ 500	0.1	Alloxan	Male albino Wister rats	[80]
*Nymphaea alba*	White water rose or white nenuphar	Nymphaeacea	Glycosides, alkaloids,phenols,tanins,flavonoids,saponin,trepenoids, phytosteroids	-	Ethanolic	200, 400	≥ 500	13	Alloxan	Albino rats	[81, 82]
*Nymphaea pubescens* Willd	Red water lily	Nymphaeacea	Alkaloids, flavonoids, glycosides,terpenoids, tannins, phenols, saponins, phytosteroids	-	Ethanolic	200, 500	≥ 200	14	Alloxan	Albino Wistar rats	[83]
*Ophiopogon japonicus*	Mondo grass	Asparagaceae	Polysaccharides	-	Aqoues	300	300	56	-	KKAy mouse	[84]
*Panax ginseng*	Ginseng	Araliaceae	Ginsenosides	-	Ethanolic	150	150	12	-	Ob/ob Mice	[85]
*Panax notoginseng*	Chinese ginseng or notoginseng	Araliaceae	Saponins	Ginsenosides, notoginsenosides	Ethanolic	50,200	≥ 50	30	-	Male kk/Ay mice	[86]
*Panax quinquefolius*	American ginseng	Araliaceae	Ginsenosides	-	Alcoholic	200	200	30–60	Streptozotoin	C57BL/6 mice	[87]
									-	db/db mice	
*Pandanus fascicularis* Lamk	Screw-pine	Pandanaceae	Saponins, tannins, phenols, alkaloids, flavonoids	-	Ethanolic	250	250	0.1	Streptozotoin	Male albino rats	[88]
*Pandanus odoratissimus*	Screwpine	Pandanaceae	Phytosteroids, phenols, isoflavones	-	Ethanolic	75, 150, 300	-	10	Alloxan	Rats	[89]
*Picrorhiza kurroa* Royle ex. Benth	Kutki	Scrophulariaceae	Cucurbitacins, polyols, phenols,iridoids,flavonoids	Picroside I and II	Alcoholic	100, 200	-	30	Streptozotocin	Male Wistar rats	[90]
*Piper longum*	Indian long pepper or pipli,pippali mula	Piperaceae	Glycosides, alkaloids	-	Aqueous	200	200	0.2	Streptozotocin	Male albino Wister rats	[91]
					Hexane	200	-				
					Ethyl acetate	200	-				
					Methanolic	200	200				
					Aqueous	200,300,400	≥ 200	30			
*Plumbago zeylanica*	Ceylon leadwort, or wild leadwort	Plumbaginaceae	-	Plumbagin	Cholorofom	15, 30	≥ 15	28	Streptozotocin	Albino Wistar rats	[92]
*Plumeria alba*	White frangipani or nosegay	Apocynaceae	-	-	Alcoholic	250	250	14	Streptozotocin	Male Sprague Dawley rats	[93]
*Potentilla fulgens* L	Bajradanti	Rosaceae	-	-	Ethanolic	100	-	30	Streptozotocin	Male Sprague Dawley rats	[94]
*Premna corymbosa* (Burm. F.) Rottl	Buas-buas	Verbenaceae	-	-	Ethanolic	200, 400	≥ 200	0.3	Alloxan	Albino Wister rats	[95]
*Quercus infectoria* Olivier	Aleppo oak	Fagaceae	-	-	Methanolic	250, 500	≥ 250	0.3	Alloxan	Albino rats	[96]
*Rauwolfia serpentina*	Indian snakeroot or devil pepper	Apocynaceae	Alkaloids, glycosides, cardiac glycosides, tannins, resins, saponins, phytosteroids, triterpenoids	-	Methanolic	10, 30, 60	≥ 10	14	Alloxan	Male Wister mice	[97]
*Rehmannia glutinosa* (Di Huang)	Chinese foxglove	Scrophulariaceae		-	-	5, 10, 20, 50	≥ 10	14	Streptozotocin	Male Wistar rats	[98]
*Rheum emodi*	Rhubarb	Polygonaceae	Anthraquinones	Emodin							
	-	2	2 mg.kg of pure Emodin	0.1	Streptozotocin	Male albino Wister rats	[99]				
*Rheum ribes* L	Rhubarb	Polygonaceae	-	Rutin, quercetin-3-D-galactoside, quercetin, fisetin, emodin, chrysophanol	Aqueous	50	50	8	Alloxan	Male Swiss-Webster mice	[100]
*Rheum turkestanicum*	Rhubarb,Rivas	Polygonaceae	-	-	Aqueous	200, 400, 600	≥ 200	21	Streptozotocin	Male Wistar rats	[101]
*Rhus mysorensis* Heyne	Mysore sumac	Anacardiaceae	Terpenoids, phytosteroids,tannins, flavonoids, Cardiac glycosides,saponins	-	Alcoholic	200, 400, 800	≥ 400	21	Streptozotocin	Male Wistar rats	[102]
*Ricinus communis*	Castor oil	Euphorbiaceae	Alkaloids, tannins, flavonoids, anthrones, saponins	-	Ethanolic	500	500	20	alloxan	Wistar rats	[103]
*Rubia cordifolia* L	Madder	Rubiaceae	-	-	Aqueous	1000	100	56	Streptozotocin	Male albino Wistar rats	[104]
*Salacia chinensis*	Saptarangi	Hippocrateaceae	Xanthonoid, phenols	Mangiferin	Isloated mangiferin	40	40	30	Streptozotocin	Male Wistar rats	[105]
*Salacia oblonga* Wall	Oblong leaf salacia	Hippocrateaceae	-	-	Hydroalcoholic	50, 100	≥ 50	94	Streptozotocin	Albino Wistar rats	[106]
*Salacia reticulata* var β-diandra	Kotalahimbatu or marking nut tree	Hippocrateaceae	-	-	Ether	233	-	0.2	Alloxan	Male Sprague–Dawley rats	[107]
					Ethyl acetate	29	-				
					Methanolic	350	350				
					Aqueous	500	-				
					Tolbutamide	15	-				
					Methanolic	175		120			
*Salvadora persica*	Miswak, toothbrush tree or mustard tree	Salvadoraceae	-	-	Hydroalcoholic	200, 400	400	21	Streptozotocin	Wister albino rats	[82]
*Sansevieria roxburghiana*	Indian bowstring heamp	Asparagaceae	Phenols, phytosteroids, fatty acids	Ferulic acid, caffeic acid, heptadecanoic acid, sinapyl alcohol, gallic acid, 4-hydroxycinnamic acid, 4-hydroxy-3-methoxybenzoic acid, protocatechuic acid, oleic acid, vanillin, hydroquinone, 4-hydroxybenzaldehyde, ergosterol, stigmasterol	Aqueous	50, 100	≥ 50	28	Streptozotocin	Wistar rats	[108]
*Sansevieria trifasciata*	Mother-in-law's tongue,Snake plant	Asparagaceae	Phenols, flavonoids, alkaloids, terpenoids, saponins, phytosteroids, glycosides	-	Methanolic	50, 100	100	15	Streptozotocin	Male Swiss albino rats	[109]
*Smilax china* L	China root	Smilacaceae	Phytosteroids, alkaloids, resin, tannin, saponins, phenols	-	Ethanolic	1000	1000	10	Alloxan	Albino rats	[110]
*Smilax moranensis* M	Cocolmecatl	Smilacaceae	-	3-*O*-caffeoyl-quinic acid, 5-*O*-caffeoyl-quinic acid & trans-resveratrol	Ethanolic	80	80	42	Streptozotocin	Wistar rats	[111]
*Sphaeranthus indicus*	East Indian globe thistle	Asteraceae	-	Gallic acid, quercetin	Ethanolic	100, 200	≥ 100	28	Streptozotocin	Wistar albino rats	[112]
*Tectona grandis* L	Teak tree	Verbenaceae	-	-	Methanolic	250, 500	≥ 250	7	Alloxan	Male albino Wister rats	[113]
*Terminalia superba*	Limba or afara	Combretaceae	-	Methyl gallate	Methanolic	200	200	14	Alloxan	Wistar rats	[114]
*Tetrapleura tetraptera*	Prekese	Fabaceae	-	-	Aqueous	150, 300	≥ 150	35	Streptozotocin	Wistar rats	[115]
*Trapa natans*	Water caltrop	Lythraceae	Flavonoids, phenols, tannins, phytosteroids	Ferulic acid, caffeic acid	Ethanolic	50, 100, 200	≥ 100	-	Streptozotocin	Wistar rats	[116]
*Trichosanthes dioica*	Chinese cucumber or snakegourd	Cucurbitaceae	-	-	Aqueous	500, 1200	-	0.1	Streptozotocin- nicotinamide	Mice	[117]
*Trichosanthes tricuspidata*	Indrayan	Cucurbitaceae	Glycosides, terpenoids	-	Ethanolic	200, 400	≥ 100	21	Alloxan	Male albino Wister rats	[118]
*Triticum repens* L. or *Agropyron repens*	Couch grass, N’jm L’bouri or outara	Poaceae	-	-	Aqueous	20	20	14	Streptozotocin	Male Wistar rats	[119]
*Withania somnifera* L	Ashwagandha, Indian ginseng or poison gooseberry	Solanaceae	Flavonoids	-	Ethanolic	100, 200	≥ 100	56	Alloxan	Male albino Wistar rats	[120]
*Xeromphis uliginosa* Retz	Bherani or pindalu	Rubiaceae	-	-	Methanolic	500	-	7	Alloxan	Evan’s Rats	[121]
*Zaleya decandra* L. N. Burm. F	Horse purslane	Aizoaceae	Flavonoids, alkaloids, phytosterol, cardic glycosides, terpenoids, tannins, phenols	-	Ethanolic	200	200	15	Alloxan	Albino Wistar rat	[122]
*Zingiber officinale*	Ginger	Zingiberaceae	-	-	Ethanolic	50,100,200,400,800	≥ 50	0.3	Treptozotocin	Wistar rats	[123]
*Ziziphus mucronata* Willd	Buffalo thorn	Rhamnaceae	-	-	Butanolic	150 or 300	300	28	Streptozotocin	Male Sprague–Dawley rats	[124]

### Phenols and flavonoids

**Fig. 3:**
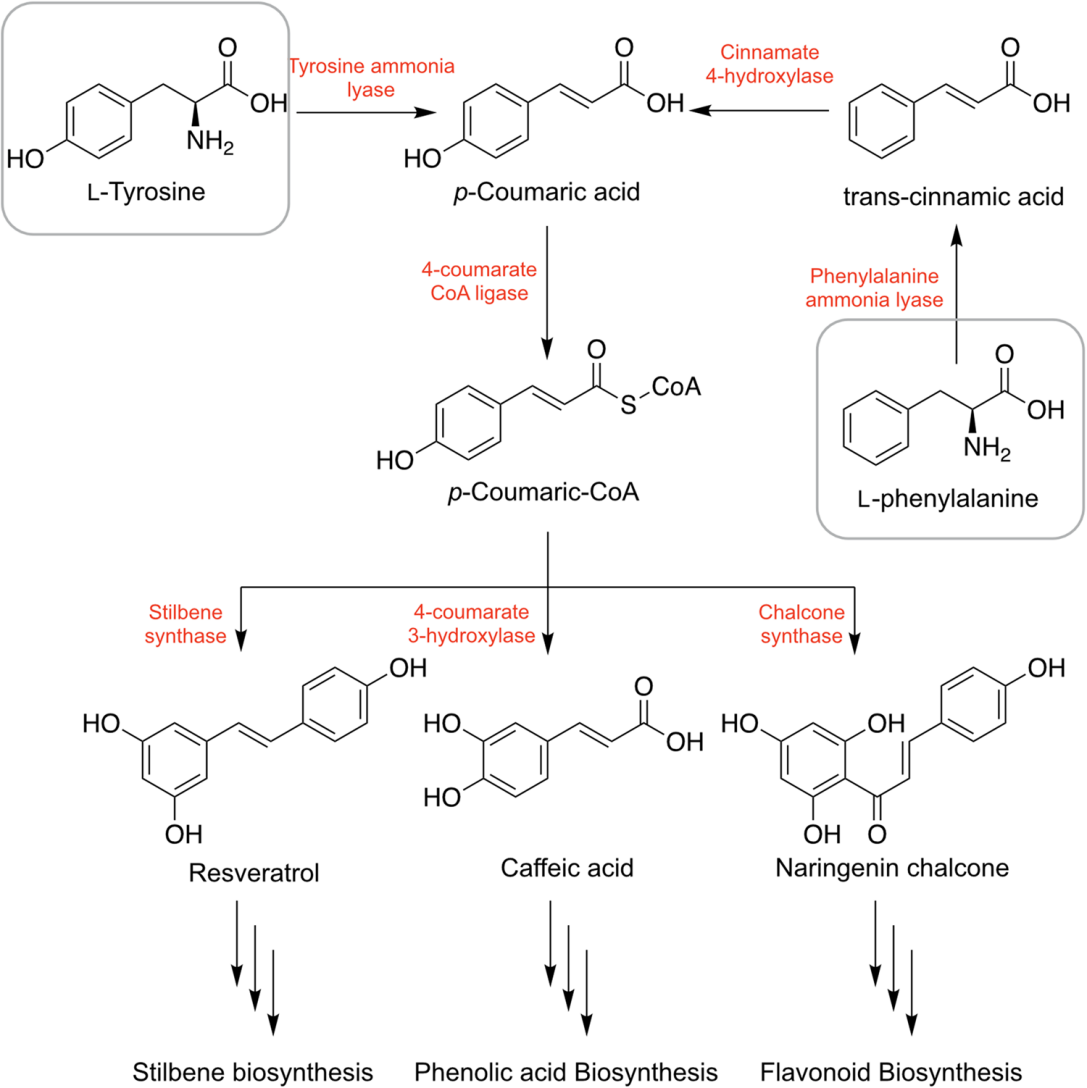
Biosynthesis pathway of phenols and flavonoids in the plant root system

Phenols constitute the largest group of natural products, with a chemical structure consisting of an aromatic ring and a hydroxyl group (C_6_H_5_OH). Within this group, flavonoids, which can be sub-categorized into flavonols, flavones, flavan-3-ols, anthocyanidins, flavanones, and isoflavones, are the largest subgroup [12]. Generally, flowers, fruits, leaves, and seeds are rich in phenols and flavonoids. However, studies have also reported phenols and flavonoids as the major chemical constituents in plant roots [125, 126]. Phenols and flavonoids are synthesized through the phenylpropanoid pathway, transforming L-phenylalanine by phenylalanine ammonia lyase or L-tyrosine by tyrosine ammonia lyase into *p*-coumaroyl-CoA, which eventually enter the phenol and flavonoid biosynthesis pathway (Fig. 3). Studies have shown plant-derived phenols, and flavonoids protect against oxidative stress, which results in improved protection against diabetes [127]. Phenols and flavonoids are furthermore well-recognized for their health benefits, including antioxidant, anti-inflammatory, antidiabetic, anti-ulcer, and anti-cancer effects [128–132].

Phenols, such as resveratrol, curcumin, chlorogenic acid, gallic acid, and ellagic acid, as well as flavonoids, such as quercetin, hesperidin, naringin, rutin, and myricetin, are well-known natural compounds for their potential antidiabetic properties. Quercetin, as one of the most abundant flavonoids in the plant kingdom, has been shown to possess several biological activities related to diabetes, such as glucose homeostasis, increased insulin sensitivity and secretion, glucose utilization in peripheral tissues, and the inhibition of intestinal glucose absorption [133, 134].

Despite promising activities in in vitro models, the low oral bioavailability of the flavonoid aglycones often results in vivo concentrations being too low to reach the relevant therapeutic concentrations [135]. Such challenges can, however, be alleviated by suitable formulations as reviewed by Zhao et al. [136].

### Alkaloids

Alkaloids cover a wide range of natural products, which are mainly found in plants [137]. Alkaloids are defined by containing a non-amide nitrogen atom in their structure [138]. Amino acids such as histidine, lysine, ornithine, tryptophan, and tyrosine are the key precursors of most alkaloids in plants. Generally, due to the pharmacological properties of the alkaloids, the primary physiological function in plant roots of this compound class is protection against herbivores. Alkaloids are widely distributed within the plant kingdom and routinely isolated from plant families such as Solanaceae, Fabaceae, Papaveraceae, Berberidaceae, and Cannabaceae. The classification of alkaloids is mainly based on either their heterocyclic ring system or the name of the plant origin. Nicotine, atropine, berberine, morphine, and caffeine are some examples of currently marketed alkaloids for the treatment of cardiovascular, inflammatory, and mental diseases [139, 140]. Alkaloids mainly possess activities related to the central nervous system as well as anti-inflammatory effects, but antidiabetic activities have also been demonstrated [11]. Particularly the benzylisoquinoline alkaloids berberine and palmatine, found in root and rhizomes of the Berberidaceae plant family, have shown promising activities for the treatment of diabetes. Lee has recently reported that isoquinoline alkaloids isolated from *Coptis japonica* showed strong antidiabetic activity as aldose reductase inhibitors in an in vivo study [141]. Chen et al. reported that berberine could potentially activate AMPK (5-adenosine monophosphate-activated protein kinase) to improve insulin sensitivity and subsequently decrease the serum glucose level [142].

### Phytosteroids

Phytosteroids are an important group of secondary metabolites produced by plants. Phytosteroids, found in plant roots in the two main forms of glycolipids and fatty acid esters [143], are involved in plant growth regulation, reproduction and respond to various biotic and abiotic stresses. The sterol primarily constitutes lipid-like molecules with intriguing antidiabetic potential. In a clinical study, Baker et al. have shown that the sterols present in vegetables, fruits, and seeds have the ability to decrease the concentration of cholesterol in diabetic patients [144]. Today, sterol-rich plant-based foods have become a focus of attention because of their enormous health benefits [145]. Nissinen et al. reported a lowering of the low-density lipoprotein (LDL) cholesterol concentrations by inhibiting cholesterol absorption in the small intestine [146], while Semova and co-workers showed that sterol-rich plant-based food enhanced the effects of antidiabetic drugs and reduced the blood glucose level [147].

### Saponins

**Fig. 4:**
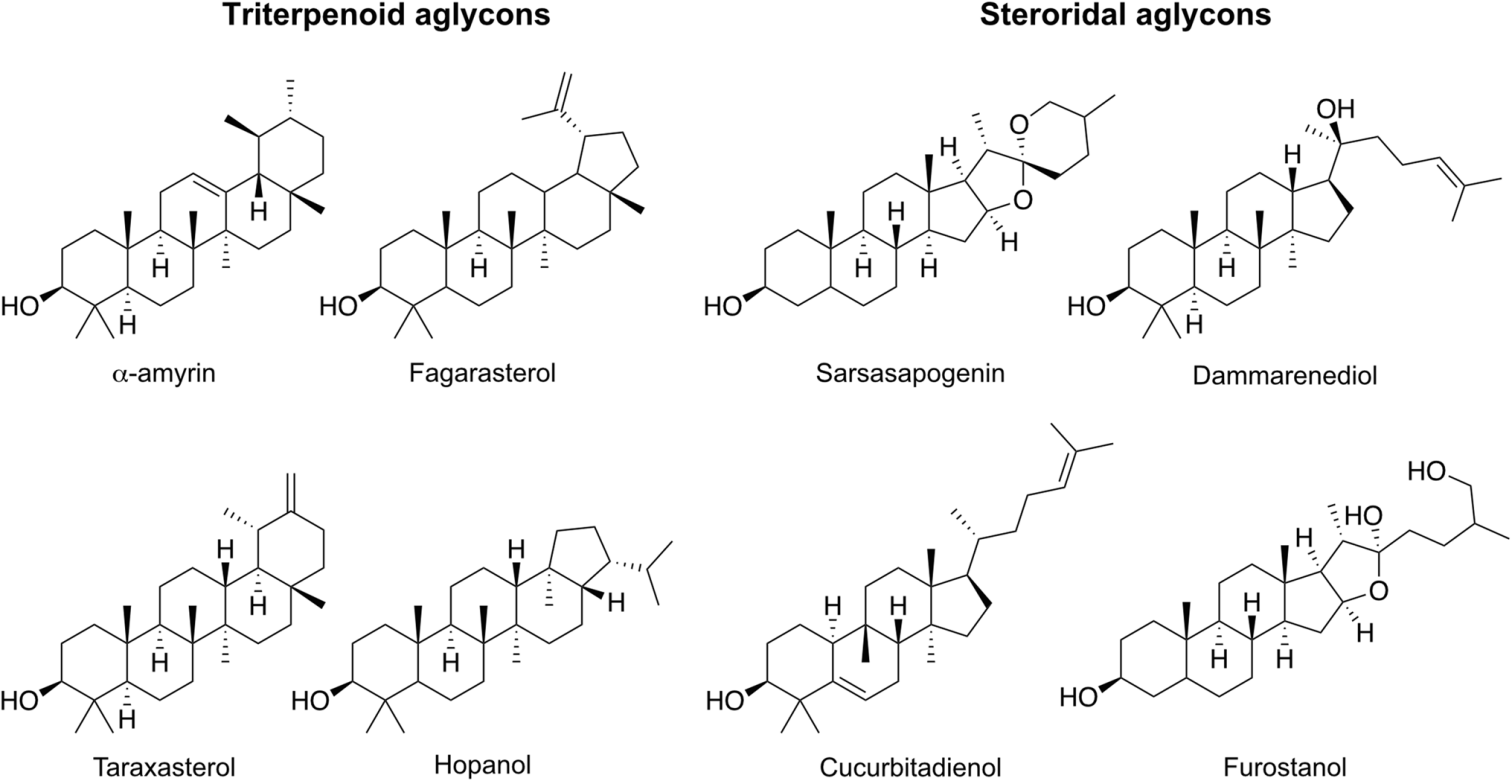
Chemical structure of selected triterpenoid and steroidal aglycones of saponins present in the plant root system

Saponins consist of triterpenoid or steroidal aglycones linked to oligosaccharide moieties (Fig. 4) and are widely distributed in the plant kingdom. These secondary metabolites are biosynthesized in leaves, flowers, and roots. Saponins have an important role in plant ecology as a defense system against pests and herbivores. Saponins are furthermore also broadly used in the food (additives), cosmetic (soaps), agricultural (pesticides), and pharmaceutical industries (production of steroid hormones) [148].

These molecules are well-known for inhibiting α-amylase, α-glucosidase enzymes, and aldose reductase, which are key enzymes for managing T2D by lowering the carbohydrate absorption in the small intestine and colon [149]. Several in vivo studies supported in vitro findings of the potential of saponins for the management of T2D. These include an investigation by Ezzat et al., which demonstrated how furostanol saponins from *Balanites aegyptiaca* reduced the blood glucose level in rats [150]. Chen et al. showed that a daily injection of saponins isolated from *P. notoginseng* resulted in a significant decrease in the blood glucose level and body mass index of male mice after 12 days [86]. Diosgenin, as the main sapogenin in *Trigonella. foenum-graecum* seeds were shown by Uemura and co-workers to decrease plasma and hepatic triglycerides in obese diabetic mice and resulted in lowered blood glucose levels [151]. Twelve triterpenoid saponins isolated from *A. taibaiensis* effectively decreased the blood glucose level, triglyceride, and Low-Density Lipoprotein-Cholesterol (LDL-C) levels in diabetic rats. Li et al. suggested that the triterpenoid saponins might activate the AMPK and can be used as an adjunctive treatment for metabolic disorders [34].

### Tannins

**Fig. 5:**
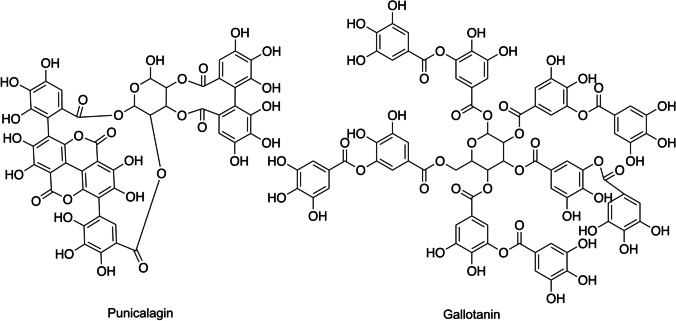
Chemical structure of hydrolyzable (punicalagin) and non-hydrolyzable (Gallotannin) tannins

In plants, the physiological role of the polyphenolic tannins is to provide protection against herbivores while also negatively affect neighboring plant growth. These secondary metabolites can be classified into hydrolyzable and non-hydrolyzable tannins. Structurally, the hydrolyzable tannins consist of a central polyhydric alcohol (often glucose) which is esterified by phenolic groups such as gallic acid (gallotannins) or hexahydroxydiphenic acid (ellagitannins) as shown in Fig. 5.

Non-hydrolyzable tannins are distinctively different from hydrolyzable tannins as they are polymerized products of flavan-3-ols and flavan-3,4-diols [152] as depicted in Fig. 5. It is well-established that tannins cause a decrease in feed intake, growth rate, feed efficiency, and protein digestibility, resulting in increased excretion of proteins and essential amino acids followed by a decrease of the body mass index [152–154]. In a study by Venkataiah et al., tannins in the root of *A. ilicifolius* were shown to significantly decrease the blood glucose level in diabetic rats when orally administering 200 mg/kg of the extract for two weeks [21]. Shokeen et al. treated normal and diabetic mice with 50% ethanolic extract of *R. communis,* which is a tannin-rich plant, daily for 20 days and showed a significant decrease in their fasting blood glucose level, total lipid profile, and liver and kidney functions [103]. Former in vitro studies have also shown that hydrolyzable tannins may inhibit the α-glucosidase activity while also slowing the starch digestion. This indicates a polypharmacological antidiabetic potential of this compound class [155, 156].

### Terpenoids

The terpenoids originate from one to several isoprene molecules (C_5_H_8_) and are widely distributed in plants and are classified based on the number of their isoprene units. The most simple class of terpenoids is the hemiterpenoids (C_5_H_8_) with additional isoprene units leading to the monoterpenoids (C_10_H_16_), sesquiterpenoids (C_15_H_24_), diterpenoids (C_20_H_32_), sesterterpenoids (C_25_H_40_), triterpenoids (C_30_H_48_), tetraterpenoids (C_40_H_64_), and polyterpenoids ([C_5_H_8_]_n_). Terpenoids are known for their antibacterial, antifungal, and anti-inflammatory bioactivity. Furthermore, in vivo and in vitro antidiabetic activities, targeting α-glucosidase, α-amylase, and protein tyrosine phosphatase have also been reported, indicating their pharmacological potential [101, 157]. Several in vivo studies show that terpenoids enhance glucose metabolism, prevent the development of insulin resistance, and normalize plasma glucose and insulin levels [158].

### Anthraquinones

**Fig. 6:**
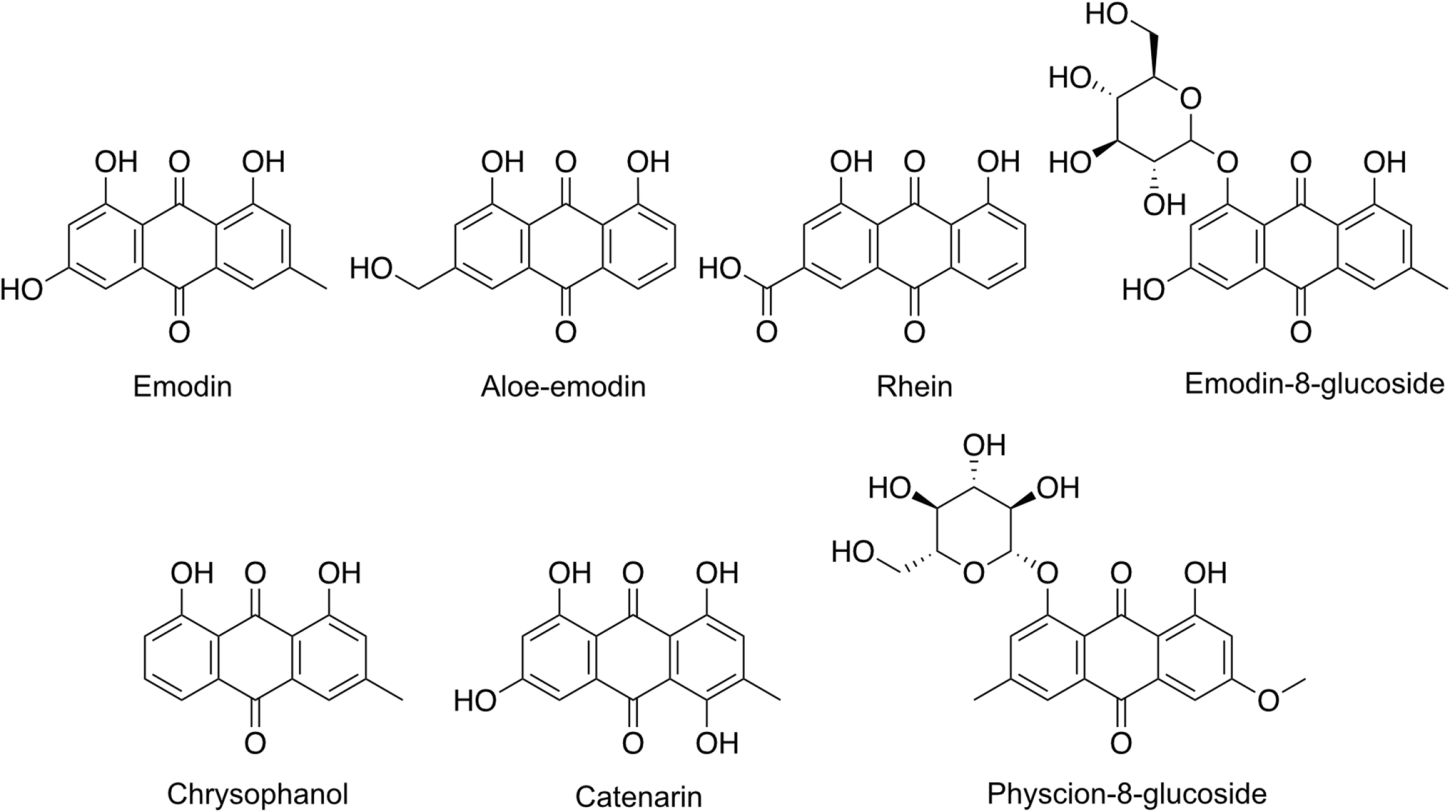
Chemical structure of the most frequent anthraquinones in the root system with α‐amylase and α‐glucosidase inhibitory activities

Anthraquinones structurally consist of two aromatic rings joined together by two carbonyl groups, creating a planar, aromatic structure. In plants, anthraquinones are synthesized through two main biosynthetic pathways: the polyketide pathway and the chorismate/*O*-succinylbenzoic acid pathway [159]. These metabolites are present in aerial parts and roots as both *O-* and *C-*glycosides as well as aglycons (Fig. 6).

Several in vivo studies have shown that anthraquinones possess activities for treatment of diabetes, suggesting this compound class as potential antidiabetic candidates [30, 41, 160]. Emodin, aloe-emodin, catenarin, chrysophanol, and rhein are the most frequently isolated aglycon anthraquinones in the root system possessing α‐amylase and α‐glucosidase inhibitory activities [160] (Fig. 6).

### Cardiac glycosides

The cardiac glycosides consist of a steroid molecule bound to one or more carbohydrates. The functional groups, which include methyl, hydroxyl, or aldehyde groups, are attached to the cardiac glycosides skeleton and play a pivotal role in the biological activity of these molecules. Cardiac glycosides enhance the heart output force and increase its rate by acting on the sodium–potassium ATPase pump [161] and are marketed for the treatment of various heart diseases. With the sodium–potassium ATPase being involved in metabolic diseases such as diabetes and obesity, regulation and enhancement of the ATPase have the potential to benefit the treatment of diabetes [161]. Several in vivo studies indicate the antidiabetic activity of cardiac glycosides present in plants [30, 59, 97].

## Conclusion

This review focuses on the literature survey of in vivo antidiabetic effects of root and rhizome extracts on streptozotocin-induced or alloxan-induced diabetic mice or rats. The literature study revealed that most of the phytochemicals with antidiabetic bioactivity in the plant root system are involved in the management of diabetes through reducing hyperglycemia and hyperlipidemia, α-glucosidase inhibition, and insulin secretion regulation. However, as in vivo studies of purified secondary metabolites from root extracts are limited, plant roots constitute a largely uninvestigated source of candidates for the treatment of diabetes. This literature review found that flavonoids, phenolic compounds, alkaloids, and phytosteroids are the most abundant chemical constituents in the root system possessing antidiabetic activities. Based on our findings, the plant families Fabaceae, Araliaceae, Asparagaceae, Asteraceae, and Zingiberaceae are considered the plant families with root extracts most likely to include natural antidiabetic compounds. As the majority of studies on antidiabetic bioactivities of plants are performed on the aerial parts, whereas root extracts are less investigated with unique natural products, the root system is a promising source of new natural compounds with antidiabetic activities. This review provides comprehensive information about the promising plants and plant families with potential antidiabetic constituents in their root system.

## References

[1] IDF Diabetes Atlas, 9th edition. Brussels, Belgium. 2019.

[2] D.P.P.R., Group (2002). Reduction in the incidence of type 2 diabetes with lifestyle intervention or metformin. N Engl J Med.

[3] Jandaghi P (2016). Lemon balm: A promising herbal therapy for patients with borderline hyperlipidemia—A randomized double-blind placebo-controlled clinical trial. Complement Ther Med.

[4] Liu Q (2014). Vitamin B12 status in metformin treated patients: systematic review. PLoS One.

[5] Marcovecchio ML, Lucantoni M, Chiarelli F (2011). Role of chronic and acute hyperglycemia in the development of diabetes complications. Diabetes Technol Ther.

[6] Israili ZH (2011). Advances in the treatment of type 2 diabetes mellitus. Am J Ther.

[7] Ardalani H (2016). Sumac as a novel adjunctive treatment in hypertension: a randomized, double-blind, placebo-controlled clinical trial. Rsc Adv.

[8] Go AS (2013). Executive summary: heart disease and stroke statistics—2013 update: a report from the American Heart Association. Circulation.

[9] Godbout A, Chiasson J-L (2007). Who should benefit from the use of alpha-glucosidase inhibitors?. Current Diab Rep.

[10] Saper RB, Eisenberg DM, Phillips RS (2004). Common dietary supplements for weight loss. Am Fam Physician.

[11] Rasouli H. et al. Anti-diabetic potential of plant alkaloids: Revisiting current findings and future perspectives. Pharmacol Res, 2020; p. 104723. 10.1016/j.phrs.2020.104723 32105756

[12] Hussain T (2020). Flavonoids and type 2 diabetes: Evidence of efficacy in clinical and animal studies and delivery strategies to enhance their therapeutic efficacy. Pharmacol Res.

[13] Newman DJ, Cragg GM (2012). Natural products as sources of new drugs over the 30 years from 1981 to 2010. J Nat Prod.

[14] Zhao Y (2019). Unraveling the complexity of complex mixtures by combining high-resolution pharmacological, analytical and spectroscopic techniques: antidiabetic constituents in Chinese medicinal plants. Faraday Discuss.

[15] Wolfender J-L (2018). Accelerating metabolite identification in natural product research: toward an ideal combination of liquid chromatography–high-resolution tandem mass spectrometry and NMR profiling, in silico databases, and chemometrics. Anal Chem.

[16] Xu L (2018). Natural products for the treatment of type 2 diabetes mellitus: Pharmacology and mechanisms. Pharmacol Res.

[17] Yadav, R. and M. Agarwala, Phytochemical analysis of some medicinal plants. J Phytology, 2011.

[18] Ee G (2009). A new anthraquinone from *Morinda citrifolia* roots. Nat Prod Res.

[19] Zhao Y (2018). Quadruple high-resolution α-glucosidase/α-amylase/PTP1B/radical scavenging profiling combined with high-performance liquid chromatography–high-resolution mass spectrometry–solid-phase extraction–nuclear magnetic resonance spectroscopy for identification of antidiabetic constituents in crude root bark of *Morus alba* L. J Chromatogr A.

[20] Zhao Y (2017). Potential of *Polygonum cuspidatum* root as an antidiabetic food: dual high-resolution α-glucosidase and PTP1B inhibition profiling combined with HPLC-HRMS and NMR for identification of antidiabetic constituents. J Agric Food Chem.

[21] Venkataiah G (2013). Anti-diabetic activity of *Acanthus ilicifolius* root extract in alloxan induced diabetic rats. Indo Am J Pharmaceutic Res.

[22] Liu Y-X (2015). Effects and molecular mechanisms of the antidiabetic fraction of *Acorus calamus* L. on GLP-1 expression and secretion in vivo and in vitro. J Ethnopharmacol.

[23] Hu, X., et al., Evaluation of anti-hyperglycemic effect of *Actinidia kolomikta* (Maxim. et Rur.) Maxim. root extract. Pakis J Pharmaceutic Sci 2015; 28. 26051735

[24] Agrawal R, Sethiya NK, Mishra S (2013). Antidiabetic activity of alkaloids of *Aerva lanata* roots on streptozotocin-nicotinamide induced type-II diabetes in rats. Pharmaceutic Biol.

[25] Rajasekar R (2014). Effect of *Alpinia calcarata* on glucose uptake in diabetic rats-an in vitro and in vivo model. J Diab Metabol Disord.

[26] Srividya A (2010). Antioxidant and antidiabetic activity of *Alpinia galanga*. Int J Pharmacognocy and Phytochem Res.

[27] Satyanand Tyagi M (2011). Antidiabetic effect of *Anacyclus pyrethrum* DC in alloxan induced diabetic rats. Eur J Biol Res.

[28] Rao NK (2006). Anti-hyperglycemic and renal protective activities of *Andrographis paniculata* roots chloroform extract. Iran J Pharmacol Ther.

[29] Miura T (2001). Antidiabetic activity of the rhizoma of *Anemarrhena asphodeloides* and active components, mangiferin and its glucoside. Biol Pharm Bull.

[30] Okokon JE, Antia BS, Udobang JA (2012). Antidiabetic activities of ethanolic extract and fraction of *Anthocleista djalonensis*. Asian Pac J Trop Biomed.

[31] Anyanwu GO (2019). Antidiabetic activities of chloroform fraction of *Anthocleista vogelii* Planch root bark in rats with diet-and alloxan-induced obesity-diabetes. J Ethnopharmacol.

[32] Jayakar B, Suresh B (2003). Antihyperglycemic and hypoglycemic effect of *Aporosa lindleyana* in normal and alloxan induced diabetic rats. J Ethnopharmacol.

[33] Ohno H (2012). Effect of aqueous extract from the root cortex of *Aralia elata* on intestinal alpha-Glucosidases and postprandial glycemic response in mice. Int J Phytomedicine.

[34] Li Y (2017). Identification of AMPK activator from twelve pure compounds isolated from *Aralia Taibaiensis*: implication in antihyperglycemic and hypolipidemic activities. Korean J Physiol Pharmacol.

[35] Adewole SO, Ojewole JA (2007). *Artocarpus communis* Forst. root-bark aqueous extract-and dtreptozotocin-induced ultrastructural and metabolic changes in hepatic tissues of wistar rats. Afr J Tradit Complement Altern Med.

[36] Vadivelan R (2011). Hypoglycemic, antioxidant and hypolipidemic activity of *Asparagus racemosus* on streptozotocin-induced diabetic in rats. Adv Appl Sci Res.

[37] Lee DH, Han JM, Yang WM (2015). The effects of *Atractylodes japonica* Koidz. on type 2 diabetic rats. J Korean Med.

[38] Patil P (2013). Antidiabetic activity of alcoholic extract of neem (*Azadirachta indica*) root bark. Natl J Physiol Pharm Pharmacol.

[39] Singh J, Kakkar P (2009). Antihyperglycemic and antioxidant effect of *Berberis aristata* root extract and its role in regulating carbohydrate metabolism in diabetic rats. J Ethnopharmacol.

[40] Gulfraz M (2007). Antihyperglycemic effects of *Berberis lyceum* Royle in alloxan induced diabetic rats. Diabetol croat.

[41] Meliani N (2011). Hypoglycaemic effect of *Berberis vulgaris* L. in normal and streptozotocin-induced diabetic rats. Asian Pac J of Trop Biomed.

[42] Alam P (2018). Anti-diabetic effect of *Boerhavia diffusa* L. root extract via free Radical scavenging and antioxidant mechanism. Toxicol and Environ Health Sci.

[43] Jung UJ (2008). Effects of the ethanol extract of the roots of *Brassica rapa* on glucose and lipid metabolism in C57BL/KsJ-db/db mice. Clin Nutri.

[44] Karimulla B, Kumar K (2011). Antidiabetic and antihyperlipidemic activity of bark of *Bruguiera gymnorrhiza* on streptozotocin induced diabetic rats. Asian J Pharm Sci Technol.

[45] Kumar R (2012). Type 2 antidiabetic activity of bergenin from the roots of *Caesalpinia digyna* Rottler. Fitoterapia.

[46] Nahar L (2014). Comparative study of antidiabetic activity of *Cajanus cajan* and *Tamarindus indica* in alloxan-induced diabetic mice with a reference to in vitro antioxidant activity. Pharmacogn Res.

[47] Prakasam A, Sethupathy S, Pugalendi KV (2003). Effect of *Casearia esculenta* root extract on blood glucose and plasma antioxidant status in streptozotocin diabetic rats. Pol J Pharmacol.

[48] Parameshwar P (2012). Hypoglycemic and anti-lipidemic effects of hydroethanolic extract of *Ceiba pentandra* Linn. Int J Pharm Appl.

[49] Hardeep F, Pandey D (2013). Anti-diabetic activity of methanolic extract of chicory roots in streptozocin induced diabetic rats. Int J Pharm.

[50] Agarwal V (2012). Hypoglycemic effects of *Citrullus colocynthis* roots. Acta Pol Pharm.

[51] Ojewole JA (2002). Hypoglycaemic effect of *Clausena anisata* (Willd) Hook methanolic root extract in rats. J Ethnopharmacol.

[52] Yuan L (2006). Hypoglycemic and hypocholesterolemic effects of *Coptis chinensis* franch inflorescence. Plant Foods Hum Nutr.

[53] Daisy P, Eliza J, Ignacimuthu S (2008). Influence of *Costus speciosus* (Koen.) Sm. Rhizome extracts on biochemical parameters in streptozotocin induced diabetic rats. J Health Sci.

[54] Madhavan V (2007). Antidiabetic activity of *Curculigo orchioides*. Root tuber Pharm Biol.

[55] Srividya AR et al. Antioxidant and antidiabetic activity of *Curcuma aromatica*. Int J of Res Ayurveda Pharm, 2012; 3(3).

[56] Mohammed A (2017). Hypoglycemic activity of *Curcuma longa* Linn root extracts on alloxan induced diabetic rats. Saudi J Life Sci.

[57] Singh P (2015). Antidiabetic activity of ethanolic extract of *Cyperus rotundus* rhizomes in streptozotocin-induced diabetic mice. J Pharm Bioallied sci.

[58] Belayneh YM (2019). Evaluation of in vivo antidiabetic, antidyslipidemic, and in vitro antioxidant activities of hydromethanolic root extract of *Datura stramonium* L. (solanaceae). J Exp Pharmacol.

[59] Nimenibo-Uadia R (2003). Control of hyperlipidaemia, hypercholesterolaemia and hyperketonaemia by aqueous extract of *Dioscorea dumetorum* tuber. Trop J pharm Res.

[60] Daisy P, Priya CE, Vargese L (2011). A study on the regenerative potential of the root and leaf extracts of *Elephantopus scaber* L.: An antidiabetic approach. Afr J of Pharm Pharmacol.

[61] Deutschländer M (2012). The hypoglycemic activity of *Euclea undulata* Thunb var myrtina (Ebenaceae) root bark evaluated in a streptozotocin–nicotinamide induced type 2 diabetes rat model. S Afr J Bot.

[62] Shahabinezhad M (2007). The effect of licorice root extract on blood sugar level in streptozotocin induced diabetic rats. J Rafsanjan Univ Med Sci.

[63] Ko, B.-S., et al., Changes in components, glycyrrhizin and glycyrrhetinic acid, in raw *Glycyrrhiza uralensis* Fisch, modify insulin sensitizing and insulinotropic actions. Biosci Biotechnol Biochem, 2007, 0705080394–0705080394. 10.1271/bbb.60533 17587675

[64] Kasiviswanath R, Ramesh A, Kumar KE (2005). Hypoglycemic and antihyperglycemic effect of *Gmelina asiatica* L INN. in normal and in alloxan induced diabetic rats. Biol Pharm Bull.

[65] Trilochana Y, Babu DJM, Rao PR (2017). The study of anti-diabetic activity of aqueous extract of root of *Gynandropsis gynandra* in diabetic rats. Indian J Res Pharma Biotech.

[66] Mahomed IM, Ojewole JA (2004). Analgesic, antiinflammatory and antidiabetic properties of *Harpagophytum procumbens* DC (Pedaliaceae) secondary root aqueous extract. Phytother Res.

[67] Venkatesh S (2010). Antihyperglycemic and hypolipidemic effects of *Helicteres isora* roots in alloxan-induced diabetic rats: a possible mechanism of action. J Nat Med.

[68] Mazumder A, Das S (2016). Study of oral hypoglycemic activity of rhizomes of *Hemidesmus indicus* R. BR. in albino wistar rats. Int J of Pharm Sci Res.

[69] Alarcon-Aguilar F (2005). Acute and chronic hypoglycemic effect of *Ibervillea sonorae* root extracts-II. J Ethnopharmacol.

[70] Barik R (2008). Antidiabetic activity of aqueous root extract of *Ichnocarpus frutescens* in streptozotocin-nicotinamide induced type-II diabetes in rats. Indian J Pharmacol.

[71] Tahir I.M. et al. Effects of methanolic and aqueous extracts of *Ipomoea batatas* L. on mineral contents level (calcium and magnesium) in alloxan-induced diabetic rats. Pak J Pharm Sci, 2018. 30393215

[72] Gulfraz M (2011). Antidiabetic activities of leaves and root extracts of *Justicia adhatoda* Linn against alloxan induced diabetes in rats. Afr J Biotechnolo.

[73] Chen X (2009). Anti-diabetic effects of water extract and crude polysaccharides from tuberous root of *Liriope spicata* var. prolifera in mice. J Ethnopharmacol.

[74] Jung YS (2016). The effects of *Lycii Radicis* cortex on inflammatory response through an oxidative stress and AGEs-mediated pathway in STZ-induced diabetic rats. J Korean Med.

[75] Arunachalam K, Parimelazhagan T (2012). Antidiabetic activity of aqueous root extract of *Merremia tridentata* (L.) Hall. f. in streptozotocin–induced–diabetic rats. Asian Pac J Trop Med.

[76] Bashir R. et al. Antidiabetic efficacy of *Mimosa pudica* (Lajwanti) root in albino rabbits. Int J Agric Biol 2013; 15(4).

[77] Singab ANB (2005). Hypoglycemic effect of Egyptian *Morus alba* root bark extract: effect on diabetes and lipid peroxidation of streptozotocin-induced diabetic rats. J Ethnopharmacol.

[78] Mallick C (2007). Antihyperglycemic effects of separate and composite extract of root of *Musa paradisiacal* and leaf of *Coccinia indica* in streptozotocin-induced diabetic male albino rat. Afr J Tradit Complement Altern Med.

[79] Antia BS, Okokon JE (2014). Phytochemical composition and antidiabetic activity of ethanol root extract of *Nauclea latifolia*. J Phytopharmacol.

[80] Sharma, V. and M.A. Pooja, Hypoglycemic activity of methanolic extracts of *Nyctanthes arbortristis* linn. root in alloxan induced diabetic rats. Int J Pharm Pharm Sci, 2011; **3**(3): p. 210–212.

[81] Mushtaq A (2017). Anti-diabetic and anti-hyperlipidemic action of aqueous ethanolic extracts of *Mentha spicata* (Leaves), *Plumeria alba* (Leaves) and *Nymphaea alba* (Flowers and Rhizomes). Int J Biol Pharm Allied Sci.

[82] Hooda MS (2014). Antihyperglycemic and antihyperlipidemic effects of *Salvadora persica* in streptozotocin-induced diabetic rats. Pharm Biol.

[83] Shajeela P, Kalpanadevi V, Mohan V (2012). Potential antidiabetic, hypolipidaemic and antioxidant effects of *Nymphaea pubescens* extract in alloxan induced diabetic rats. J Appl Pharm Sci.

[84] Wang L-Y (2012). MDG-1, a polysaccharide from *Ophiopogon japonicus* exerts hypoglycemic effects through the PI3K/Akt pathway in a diabetic KKAy mouse model. J Ethnopharmacol.

[85] Dey L (2003). Anti-hyperglycemic effects of ginseng: comparison between root and berry. Phytomedicine.

[86] Chen Z-H (2008). Saponins isolated from the root of *Panax notoginseng* showed significant anti-diabetic effects in KK-Ay mice. Am J Chinese Med.

[87] Sen S, Querques MA, Chakrabarti S (2013). North American ginseng (*Panax quinquefolius*) prevents hyperglycemia and associated pancreatic abnormalities in diabetes. J Med Food.

[88] Rajeswari J, Kesavan K, Jayakar B (2012). Antidiabetic activity and chemical characterization of aqueous/ethanol prop roots extracts of *Pandanus fascicularis* Lam in streptozotocin-induced diabetic rats. Asian Pac J Trop Biomed.

[89] Venkatesh S (2012). Antidiabetic activity of *Pandanus odoratissimus* root extract. Indian J Pharm Educ.

[90] Kumar S (2017). *Picrorhiza kurroa* enhances β-Cell mass proliferation and insulin secretion in streptozotocin evoked β-Cell damage in rats. Front Pharmacol.

[91] Nabi SA (2013). Antidiabetic and antihyperlipidemic activity of *Piper longum* root aqueous extract in STZ induced diabetic rats. BMC complement Altern Med.

[92] Sunil, C., et al., Antidiabetic effect of plumbagin isolated from *Plumbago zeylanica* L. root and its effect on GLUT4 translocation in streptozotocin-induced diabetic rats. Food Chem Toxicol, 2012; **50**(12): p. 4356–4363. 10.1016/j.fct.2012.08.046 22960630

[93] Kadébé ZT (2016). Antidiabetic activity of *Plumeria alba* Linn (apocynaceae) root extract and fractions in streptozotocin-induced diabetic rats. Trop J Pharm Res.

[94] Pal S. et al. Antidiabetic potential of *Potentilla fulgens* roots in validated animal models of diabetes. Braz Arch Biol Technol, 2016; 59.

[95] Dash GK, Patro CP, Maiti A (2005). A study on the anti-hyperglycaemic effect of *Premna corymbosa* Rottl. roots. J Nat Med.

[96] Saini R, Patil S (2012). Anti-diabetic activity of roots of *Quercus infectoria* Olivier in alloxan induced diabetic rats. Int J Pharm Sci Res.

[97] Azmi MB and Qureshi SA, Methanolic root extract of *Rauwolfia serpentina* benth improves the glycemic, antiatherogenic, and cardioprotective indices in alloxan-induced diabetic mice. Adv Pharmacol Sci 2012. 10.1155/2012/376429 PMC3535824 23365565

[98] Zhu H (2016). Antidiabetic and antioxidant effects of catalpol extracted from *Rehmannia glutinosa* (Di Huang) on rat diabetes induced by streptozotocin and high-fat, high-sugar feed. Chin Med.

[99] Arvindekar A (2015). Evaluation of anti-diabetic and alpha glucosidase inhibitory action of anthraquinones from *Rheum emodi*. Food Funct.

[100] Raafat K, El-Lakany A (2017). Combination of *Rheum ribes* and metformin against diabetes, thermal hyperalgesia, and tactile allodynia in a mice model. Altern Ther Health Med.

[101] Mousa-Al-Reza Hadjzadeh ZR (2017). *Rheum turkestanicum* rhizomes possess anti-hypertriglyceridemic, but not hypoglycemic or hepatoprotective effect in experimental diabetes. Avicenna J Phytomed.

[102] Mal Lamba S et al., Anti-diabetic, hypolipidemic and anti-oxidant activities of hydroethanolic root axtract of *Rhus Mysurensis* heyne in streptozotocin induced diabetes in wistar male rats. Pharmacogn J, 2014; 6(3).

[103] Shokeen P (2008). Antidiabetic activity of 50% ethanolic extract of *Ricinus communis* and its purified fractions. Food Chem oTxicol.

[104] Baskar R (2006). Antihyperglycemic activity of aqueous root extract of *Rubia cordifolia* in streptozotocin-induced diabetic rats. Pharm Biol.

[105] Sellamuthu PS (2014). Beneficial effects of mangiferin isolated from *Salacia chinensis* on biochemical and hematological parameters in rats with streptozotocin-induced diabetes. Pak J Pharm Sci.

[106] Bhat BM (2012). Antidiabetic and hypolipidemic effect of *Salacia oblonga* in streptozotocin induced diabetic rats. J Clin Diagn Res.

[107] Ruvin Kumara N, Pathirana R, Pathirana C. Hypoglycemic activity of the root and stem of *Salacia reticulata* var. β-diandra. in alloxan diabetic rats. Pharm Biol 2005; 43(3): p. 219–225.

[108] Bhattacharjee N. et al. *Sansevieria roxburghiana* Schult. & Schult. F.(Family: Asparagaceae) attenuates type 2 diabetes and its associated cardiomyopathy. PloS one, 2016; 11(11). 10.1371/journal.pone.0167131 PMC5125675 27893829

[109] Dey B (2014). Mechanistic explorations of antidiabetic potentials of *Sansevieria trifasciata*. Indo Glob J Pharm Sci.

[110] Bhati R (2011). Pharmacognostical standardization, extraction and anti-diabetic activity of *Smilax china* L. rhizome. Asian J Tradit Med.

[111] Romo-Pérez A, Escandón-Rivera SM, Andrade-Cetto A (2019). Chronic hypoglycemic effect and phytochemical composition of *Smilax moranensis* roots. Rev Bras Farmacogn.

[112] Ramachandran S. et al. Investigation of antidiabetic, antihyperlipidemic, and in vivo antioxidant properties of *Sphaeranthus indicus* Linn. in type 1 diabetic rats: an identification of possible biomarkers. Evid Based Complement Alternat Med, 2011. 10.1155/2011/571721 PMC2952313 20953435

[113] Pooja VS, Samanta K (2011). Hypoglycemic activity of methanolic extract of *Tectona grandis* linn. root in alloxan induced diabetic rats. J Appl Pharm Sci.

[114] Oluwarotimi Comfort D (2019). Methyl gallate from the anti-hyperglycaemic fraction of the aoot bark extract of *Terminalia Superba*. Int J Plant Stu.

[115] Omonkhua A (2014). Effect of aqueous root bark extract of *Tetrapleura tetraptera* (Taub) on blood glucose and lipid profile of streptozotocin diabetic rats. Nig Q J Hosp Med.

[116] Kharbanda C (2014). *Trapa natans* L. root extract suppresses hyperglycemic and hepatotoxic effects in STZ-induced diabetic rat model. J Ethnopharmacol.

[117] Lo H-Y (2017). Hypoglycemic effects of *Trichosanthes kirilowii* and its protein constituent in diabetic mice: the involvement of insulin receptor pathway. BMC complement Altern Medicine.

[118] Kulandaivel S, Bajpai P, Sivakumar T (2013). Antihyperglycemic activity of *Trichosanthes tricuspidata* root extract. Bangladesh J Pharmacol.

[119] Eddouks M, Maghrani M, Michel J (2005). Hypoglycaemic effect of *Triticum repens* P. Beauv. in normal and diabetic rats. J Ethnopharmacol.

[120] Udayakumar R (2009). Hypoglycaemic and hypolipidaemic effects of *Withania somnifera* root and leaf extracts on alloxan-induced diabetic rats. Int J Mol Sci.

[121] Khan MF (2015). In vivo hypoglycemic and alloxan induced antidiabetic activity of *Xeromphis uliginosa* Retz. Afr J Pharm Pharmacol.

[122] Meenakshi P (2010). Antidiabetic activity of ethanolic extract of *Zaleya decandra* in alloxan-induced diabetic rats. Appl Biochem Biotechnol.

[123] Ojewole JA (2006). Analgesic, antiinflammatory and hypoglycaemic effects of ethanol extract of *Zingiber officinale* (Roscoe) rhizomes (Zingiberaceae) in mice and rats. Phytother Res.

[124] Ibrahim MA, Islam MS (2017). Effects of butanol fraction of *Ziziphus mucronata* root ethanol extract on glucose homeostasis, serum insulin and other diabetes-related parameters in a murine model for type 2 diabetes. Pharm Biol.

[125] Zainol M (2003). Antioxidative activity and total phenolic compounds of leaf, root and petiole of four accessions of *Centella asiatica* (L.) Urban. Food Chem.

[126] Fernand VE (2008). Determination of pharmacologically active compounds in root extracts of *Cassia alata* L. by use of high performance liquid chromatography. Talanta.

[127] Ishige K, Schubert D, Sagara Y (2001). Flavonoids protect neuronal cells from oxidative stress by three distinct mechanisms. Free Radic Bio Med.

[128] Ardalani H, Hadipanah A, Sahebkar A (2020). Medicinal plants in the treatment of peptic ulcer disease: A review. Mini Rev Med Chem.

[129] Fusi F. et al. The beneficial health effects of flavonoids on the cardiovascular system: focus on K+ channels. Pharmacol Res 2020, 104625. 10.1016/j.phrs.2019.104625 31918018

[130] Raffa D (2017). Recent discoveries of anticancer flavonoids. Eur J Med Chem.

[131] Cushnie TT, Lamb AJ (2005). Antimicrobial activity of flavonoids. Int J Antimicrob Agents.

[132] Apaya MK et al. Phytochemicals as modulators of β-cells and immunity for the therapy of type 1 diabetes: Recent discoveries in pharmacological mechanisms and clinical potential. Pharmacol Res 2020, 104754. 10.1016/j.phrs.2020.104754 32173584

[133] M Eid H, Haddad PS. The antidiabetic potential of quercetin: underlying mechanisms. Curr Med Chem 2017, 24(4), 355–364. 10.2174/0929867323666160909153707 27633685

[134] AL-Ishaq, R.K., et al., Flavonoids and their anti-diabetic effects: cellular mechanisms and effects to improve blood sugar levels. Biomolecules 2019, 9(9), 430. 10.3390/biom9090430 PMC6769509 31480505

[135] Zhang L, Zuo Z, Lin G (2007). Intestinal and hepatic glucuronidation of flavonoids. Mol Pharm.

[136] Zhao J, Yang J, Xie Y (2019). Improvement strategies for the oral bioavailability of poorly water-soluble flavonoids: An overview. Int J Pharm.

[137] Roberts MF, Strack D, Wink M. Biosynthesis of alkaloids and betalains. Annu Plant Rev Online 2018, 20–91.

[138] Knolker H-J. The Alkaloids. 2017; Academic Press.

[139] Gomes JP, Watad A, Shoenfeld Y (2018). Nicotine and autoimmunity: The lotus’ flower in tobacco. Pharmacol Res.

[140] Habtemariam S. Berberine pharmacology and the gut microbiota: A hidden therapeutic link. Pharmacol Res 2020, 104722. 10.1016/j.phrs.2020.104722 32105754

[141] Lee H-S (2002). Rat lens aldose reductase inhibitory activities of *Coptis japonica* root-derived isoquinoline alkaloids. J Agric Food Chem.

[142] Chen K (2014). Berberine reduces ischemia/reperfusion-induced myocardial apoptosis via activating AMPK and PI3K–Akt signaling in diabetic rats. Apoptosis.

[143] Ferrer A (2017). Emerging roles for conjugated sterols in plants. Prog Lipid Res.

[144] Baker WL, Baker EL, Coleman CI (2009). The effect of plant sterols or stanols on lipid parameters in patients with type 2 diabetes: a meta-analysis. Diabetes Res Clin Prac.

[145] Marangoni F, Poli A (2010). Phytosterols and cardiovascular health. Pharmacol Res.

[146] Nissinen M (2002). Micellar distribution of cholesterol and phytosterols after duodenal plant stanol ester infusion. Am J Physiol Gastrointest Liver Physiol.

[147] Semova I (2019). Type 1 diabetes is associated with an increase in cholesterol absorption markers but a decrease in cholesterol synthesis markers in a young adult population. J Clin Lipodol.

[148] Balandrin M.F. Commercial utilization of plant-derived saponins: an overview of medicinal, pharmaceutical, and industrial applications. Saponins used in traditional and modern medicine 1996, 1–14. 10.1007/978-1-4899-1367-8_1 8957279

[149] Gong X. et al. The interactions between gut microbiota and bioactive ingredients of traditional chinese medicines: A review. Pharmacol Res 2020, 104824. 10.1016/j.phrs.2020.104824 32344049

[150] Ezzat, S.M., A. Abdel Motaal, and S.A.W. El Awdan, In vitro and in vivo antidiabetic potential of extracts and a furostanol saponin from *Balanites aegyptiaca*. Pharm Biol 2017; 55(1): p. 1931–1936. 10.1080/13880209.2017.1343358 PMC6130680 28659002

[151] Uemura T (2011). Diosgenin, the main aglycon of fenugreek, inhibits LXRα activity in HepG2 cells and decreases plasma and hepatic triglycerides in obese diabetic mice. J Nutr.

[152] Chung K-T (1998). Tannins and human health: a review. Crit Rev Food Sci Nutr.

[153] Salunkhe D (1983). Chemical, biochemical, and biological significance of polyphenols in cereals and legumes. Crit Rev Food Sci Nutr.

[154] Ardalani H (2020). The effect of *Cynara scolymus* on blood pressure and BMI in hypertensive patients: A randomized, double-blind, placebo-controlled, clinical trial. Complement Med Res.

[155] Li K (2018). Persimmon tannin decreased the glycemic response through decreasing the digestibility of starch and inhibiting α-amylase, α-glucosidase, and intestinal glucose uptake. J Agric Food Chem.

[156] Yuan T (2013). New phenolics from the flowers of Punica granatum and their in vitro α-glucosidase inhibitory activities. Planta Med.

[157] Kjaerulff L (2020). Isolation, structure elucidation and PTP1B inhibitory activity of serrulatane diterpenoids from the roots of *Myoporum insulare*. Phytochem Lett.

[158] Nazaruk J, Borzym-Kluczyk M (2015). The role of triterpenes in the management of diabetes mellitus and its complications. Phytochem Rev.

[159] Han Y-S, Van der Heijden R, Verpoorte R (2001). Biosynthesis of anthraquinones in cell cultures of the Rubiaceae. Plant Cell Tissue Organ Cult.

[160] Mohammed A (2020). Antidiabetic potential of anthraquinones: A review. Phytother Res.

[161] Patel S (2016). Plant-derived cardiac glycosides: Role in heart ailments and cancer management. Biomed Pharmacother.

